# Comparison of the Uptake of Tire Particles via Suspension and Surface Deposit Feeding in the Estuarine Amphipod *Corophium volutator*

**DOI:** 10.1021/acs.est.5c03654

**Published:** 2025-08-20

**Authors:** Charlotte Woodhouse, Penelope K. Lindeque, Tamara Galloway, Geoffrey D. Abbott, Matthew Cole

**Affiliations:** † 61564 Plymouth Marine Laboratory, Prospect Place, The Hoe, Plymouth PL1 3DH, U.K.; ‡ 3286 University of Exeter, Geoffrey Pope Building, Stocker Road, Exeter EX4 4QD, U.K.; § 5994 Newcastle University, Drummond Building, Newcastle upon Tyne NE1 7RU, U.K.

**Keywords:** Tyre Particles, Interactions, Adherence, Ingestion, Microplastic, Estuarine, Invertebrate

## Abstract

Tire particles have been reported as a major source of microplastic pollution for aquatic environments, but interactions between biota and tire particles remain uncertain. In this study, we exposed the estuarine amphipod *Corophium volutator* to environmentally relevant concentrations of tire particles to quantify the ingestion and adherence of tire particles via two different feeding modes: suspension feeding and surface deposit feeding. *C. volutator* were placed into exposure treatments relevant to each feeding mode, dosed with tire particles (0.1 g/L). In both treatments, tire particles were found to be adhered and ingested by all individuals. In the suspension feeding treatment, individuals ingested significantly higher numbers of tire particles compared to the surface deposit treatment and controls (GLMM, *p* < 0.001). *C. volutator* had significantly higher numbers of adhered particles to the antenna compared to other body parts (Kruskal–Wallis, df = 6, *p* < 0.001). The impact of anthropogenic particle adherence upon biota is poorly elucidated, but an array of adverse outcome pathways are postulated based on existing literature. The outcomes of this study will help to elucidate the exposure of biota to tire particles in benthic estuarine and coastal environments.

## Graphical abstract

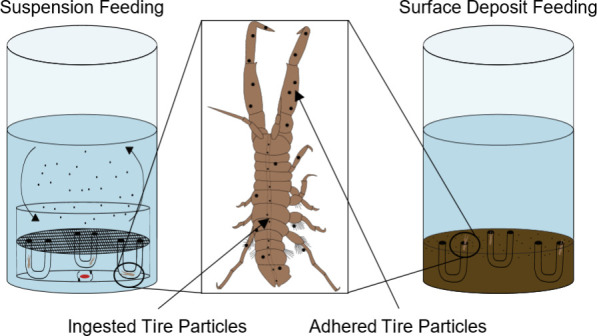

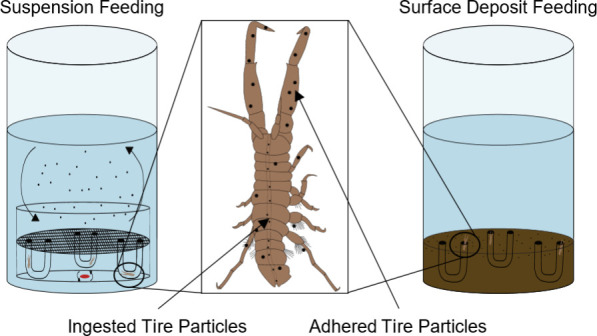

## 1 Introduction

### 1.1 Tire Particles

Tire particles, produced from abrasion between vehicle tires and roads, have been reported as a major source of particulate pollution.^1^ Tires comprise natural and synthetic rubber imbued with a range of chemicals such as polycyclic aromatic hydrocarbons including benzo­[a]­pyrene,^2^ trace metals (e.g., zinc, copper, and lead),^3^ volatile organics (e.g., benzothiazole)^4^ and antioxidants (e.g., *N*-(1,3-dimethylbutyl)-*N*′-phenyl-p-phenylenediamine (6PPD)^5^) designed to increase longevity, reduce road resistance, and make the tires UV-resistant.^6–9^ Tire particles are a type of microplastic, given they are primarily made up of synthetic rubber, solid in state, insoluble, and <5 mm in size.^10^ Several studies have indicated tire particles are the predominant source of secondary microplastics in the natural environment.^1,6,11^

As reliance on motor vehicles has increased, so too has the number of tires produced, with current production standing at 2.35 billion tires per annum across the globe.^12^ In 2019, total vehicle traffic was recorded at 573.7 billion vehicle kilometers, which was the highest it has ever been with a 2.2% increase on 2018 data and a 36.1% increase from 1994.^13^ Passenger vehicles on average produce 50–132 mg of tire particles/vehicle/km,^14^ with annual emissions of tire wear being released into the environment varying between 0.2–5.5 kg tire tread/capita/year.^7^ Across the developed world estimates for the number of tire particles released into the environment exceeds 6 million tonnes per annum.^15^

Tire particles were first recognized as an environmental pollutant in the 1970s,^16^ but received little attention over the following 50 years, leaving knowledge gaps relating to environmental concentrations, distributions, and toxicity.^1^ Since 2018, tire particles—and amalgams of tire, vehicular and road particles termed “tire road wear particles” (TRWPs)—have garnered renewed attention as a subcategory of microplastic pollution.^17–19^ Given their small size and complex chemistry, identifying and quantifying tire particles in the natural environment is extremely challenging. Unlike other types of microplastics, Fourier transform–infrared spectroscopy (FTIR) and Raman spectroscopy cannot be used to characterize tire particles owing to the high levels of carbon black that absorb infrared light.^20^ Many quantitative studies use pyrolysis-gas chromatography–mass spectrometry (Py-GC-MS) to estimate the quantities of tire particles in the environment using chemical markers associated with tires,^21^ including benzothiazoles (e.g., 24 MoBT) and styrene–butadiene rubber (SBR).^7,22^ For example, 24 MoBT was used as a reference chemical to estimate a concentration of 117 g/kg tire particles in soil 0 m from the road,^23^ and 1.6 mg/L in river waters.^24^ More recently, zinc has been used to estimate 3.2 g/kg of tire particles in stormwater settling pond sediment,^25^ and benzothiazole has been used to estimate 3.6 mg/L in a highway settling pond.^22^ Further, tire particles were identified, based upon morphological characteristics, in sediment at both the inlet and outlet of a floating treatment wetland making up 15–38% of the microplastics found in the samples.^26^ Tire particles have also been found in high abundances in intertidal estuaries making up 73% of detected microplastics with concentrations varying from below the limit of detection up to 85 g/kg.^27^ Recent studies have shown tire particles concentrations to between 0.0023–1.6 g/kg in riverine and estuarine sediments.^28,29^ However, environmental concentrations based on markers may overestimate the concentrations of tire particles found in environmental compartments due to the markers being associated with other chemicals such as antifreeze products.^29^

Pathways for tire particles entering the natural environment include air dispersal of smaller particles (<10 μm)^6^ and runoff, whereby particles are transported by rainwater into drainage systems, including settling ponds or municipal wastewater systems, or into adjacent terrestrial and aquatic ecosystems.^6,31^ In waterways, tire particles have been demonstrated to both settle in sediment owing to their relatively high densities (depending on tire type, the densities of the particles can vary from 1.2 g/cm^3^ in tire particles up to 1.8 g/cm^3^ on tire road wear particles)^26,32,33^ or be transported downstream in flowing water.^26^ The hydrodynamics of estuaries (e.g., a widening river basin causing reduced hydrodynamic flow, and salinity gradients) can result in the flocculation and settlement of anthropogenic particulates.^34–36^

### 1.2 Toxicity

Laboratory studies have provided evidence that tire particles can be ingested by *Daphnia magna,* ^37^ fish (*Fundulus heteroclitus*; *Pimephales promelas*),^38^ and an array of estuarine and brackish species, including amphipods (e.g., *Hyalella azteca*, *Gammarus pulex*,^39,40^ opossum shrimps,^41^ the clam *Scrobicularia plana*,^42^ and the polychaete *Hediste diversicolor*.^42^ Uptake of tire particles was proven by observing tire particles in the gastrointestinal tract for the mummichogs, fathead minnows,^38^ and *Daphnia magna,* ^39^ while other studies used digestion of the organisms followed by counting of the tire particles.^40,42^

Gauging the ecotoxicological risk posed by tire particles is complicated by the heterogeneity of these particulates which can range in source, shape, size, and chemical composition. To date, toxicity testing has primarily focused on tire leachates, describing the complex cocktail of metals and chemicals released from tire particles in a given media (e.g., intestinal fluids, porewater, freshwater, seawater).^37,43^ These studies have demonstrated that tire leachates can cause cellular toxicity in mussels (*Mytilus galloprovincialis*),^44,45^ altered swimming behavior of copepods, shrimp and fish (*Limnocalanus macrurus*, *Americamysis bahia*, *Menidia beryllina*),^46,47^ and lethal effects on copepods (*Calanus sp*, *Acartia longiremis*),^48^ trout (*Oncorhynchus mykiss*),^49^ and *Daphnia magna.* ^50^ More recent, toxicity studies have demonstrated that ingestion of tire particles can cause deleterious impacts in amphipods^39^ and fish.^38,47^

### 1.3 Corophium volutator

Estuaries are ecologically valuable habitats, providing nutrition and refuge to invertebrates, fish, and wading birds,^34^ and supporting ecosystem services, including habitat protection and water filtration.^51,52^ The sediment-dwelling amphipod, *Corophium volutator,* are found primarily in temperate estuarine environments in Europe and North America.^53–55^ *C. volutator* are a keystone species within estuaries owing to their roles as a bioturbator,^56^ and as a source of prey for a range of organisms including Brown shrimp (*Crangon crangon*), green shore crab (*Carcinus maenas*), and flounder (*Platichthys flesus*).^55,57,58^ *C. volutator* feed upon diatoms, bacteria, and particulate organic matter (4–63 μm)^59,60^ and demonstrate plasticity in their feeding strategy,^61^ with the capacity to capture food via either suspension or surface deposit feeding. *C. volutator* primarily feeds via surface deposit feeding, whereby the amphipods partially emerge from their burrows and use their second antennae to rake particles from the top layer of the surrounding surface sediment into their burrow.^57,62^ Suspension feeding can occur at high tide when waterborne food concentrations are high,^63^ whereby *C. volutator* use their gnathopods to create a feeding current that draws suspended particulates into their burrow.^57^

### 1.4 Aims and Hypothesis

In this study, the estuarine amphipod *Corophium volutator* is exposed to environmentally relevant concentrations of tire particles (0.1 g/kg) to quantify the adherence and ingestion of tire particles via two different feeding modes: suspension feeding and surface deposit feeding. It is hypothesized that *C. volutator* will ingest tire particles, within their optimal prey size range, via both feeding modes. The study will elucidate the extent to which sediment-dwelling invertebrates are exposed to tire particles.

## 2 Materials and Methods

### 2.1 Tire Particles

Tire particles, derived from end-of-life passenger car tire treads using high-pressure waterjet technology, were procured from a commercial supplier. Tire particles were size fractioned using a sieve shaker (RETSCH AS 200 Basic) to attain particles ≤ 150 μm (for size distributions, refer to Figure S1), which is similar in size to sediment particles ingested by *Corophium volutator.* ^60^ To prevent particle aggregation and reduce hydrophobicity, tire particles were coated in surfactant per the protocol of Ziajahromi et al.^64^ In brief: the required mass of tire particles were prepared in a solution containing 20 mL of 25 ppt salinity filtered seawater (0.2 μm Whatman glass fiber filter, FSW) and 20 μL polysorbate-80 (0.1% v/v), and vortexed for 2 min. To account for the surfactant in control treatments, a surfactant solution, absent of tire particles, was also prepared.

### 2.2 Animal Sampling and Handling

*Corophium volutator* were collected in May 2023 from the River Avon, Devon, UK (50.305504, −3.8507650), a rural river, and transported back to Plymouth Marine Laboratory in cool boxes. In the laboratory, *C volutator* were acclimated for 14 days in 2 L of aquaria containing 200 g of sieved natural sediment and 800 mL of continuously aerated FSW, held at 15 °C (±1 °C) with a 12:12 light: dark photoperiod. *Corophium* were fed once a week with 5 mL of a microalgal feedstock (123 mg/L algal biomass, see Section 2.3). Algal biomass content of the preprepared microalgal feedstock was calculated by the weight of the dried and ashed sample. The day after feeding, a 75% water change was carried out.

### 2.3 Algal Biomass

The microalgal feedstock was prepared by adding 2 mL of a commercially available blend of microalgae (Shellfish Diet 1800, Reed Mariculture) in 1 L FSW. The biomass of microalgae in the feedstock was determined as follows: 0.2 μm glass fiber filters (GFF, Whatman, n = 3) were placed in a muffle furnace (500 °C) for 12 h and then weighed immediately (Sartorius R200D, 5 decimal places). Next, 15 mL of the feedstock was vacuum-filtered onto glass filters. Filters were placed in a drying oven (60 °C) for 48 h and weighed to ascertain the dry weight (*dw*; mg) of the feedstock. The filters were then placed in a muffle furnace (500 °C) for 12 h and immediately weighed to ascertain the ash-free dry weight (*afdw*; mg). The biomass concentration (mg/L) in the feedstock was calculated as

Biomass=(dw−afdw/15)×1000

### 2.4 Sediment

Estuarine sediments were collected alongside *C. volutator*. The site is located next to a slow speed tidal road and no motorways within 10 miles (Figure S2). In the laboratory, sediments were sieved through 500 μm mesh sieves to remove the large organic matter and macrofauna. Sediments were allowed to settle overnight, and then, excess seawater siphoned off. Sediments were stored in the dark at 4 °C prior to use in the experiment.^65^

To determine the tire particle concentration in sediments, sediment samples were taken in triplicate (stored in acid-washed and muffle furnaced 25 mL glass vials) and analyzed with Py-GC-MS using 1,3-benzothiazole which is a pyrolysis product from benzothiazole-based vulcanization accelerators for tire tread wear as detailed in Parker-Jurd et al.^30^

### 2.5 Exposure 1: Suspension Feeding

To facilitate suspension feeding, *Corophium* were placed in artificial burrows. Adapting the methods of Icely and Nott,^66^ 68 mm lengths of 3 mm diameter polybutylene terephthalate (PBT) plastic tubing were molded to mimic the U-shaped burrows created by *C. volutator*. Artificial burrows were embedded, in triplicate, within 500 μm nylon mesh sieves (polycarbonate casing; 74 mm diameter, 53 mm height) designed so that the sieve could sit within a 1 L glass beaker with a magnetic flea positioned below (Figure 1A). Prior to experimental use, several drops of FSW and a single *C. volutator* was added to each of the three artificial burrows in each beaker (3 individuals per beaker and 5 beakers per treatment, n = 15). To prevent the *C. volutator* from leaving the artificial burrows, tubes were capped with 1 mm nylon mesh mounted on 8 mm lengths of 6 mm diameter silicone tubing. Next, 335 mL of FSW, 5 mL of feedstock, and 20 mL of the tire particle solution (or equivalent control) was added to the beaker to attain a waterborne concentration of 0.1 g/L (∼359,800 tire particles/L; Section 2.11) tire particles. Beakers were placed on a multiposition magnetic stirrer (Variomag, 105 rpm), with 32 mm magnetic fleas facilitating the continual suspension of tire particles throughout the beaker. To replicate the darkness of natural burrows, the setup was covered with an opaque box. After 1 h exposure, the *C. volutator* and any faeces present within their artificial burrows were carefully removed via flushing into an Eppendorf tube using FSW. Specimens were frozen at −20 °C prior to analysis.

**Figure 1:**
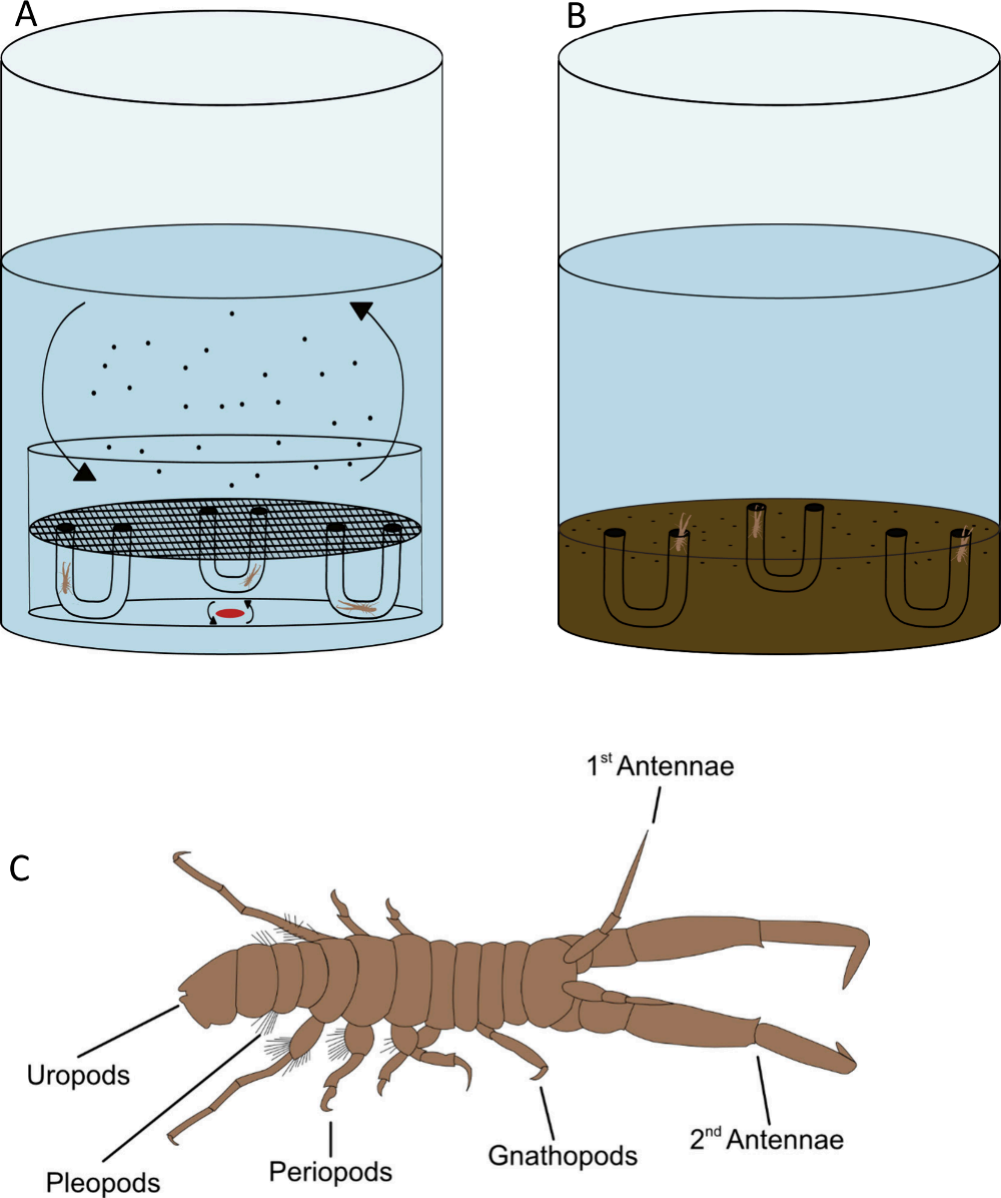
Set up of the tire particle exposure experiment. (A) represents the suspension feeding treatment (0.1 g/L tire particles) and (B) represents the surface-deposit feeding treatment where the upper layer of sediment was dosed (0.1 g/kg dw). Labeled diagram of *Corophium volutator* (C).

### 2.6 Exposure 2: Surface Deposit Feeding

To facilitate surface deposit feeding, *Corophium* were permitted to generate natural burrows in sediment in which the top 4 mm contained tire particles or equivalent control. For each replicate, 173 g of sediment was added to a 1L glass beaker, and then 150 mL of FSW carefully added, before leaving the system to settle for 2 h. Next, 52.8 g of spiked sediment, containing tire particles (0.1 g/kg w/w; ∼82,500 tire particles/kg; Section 2.11) or equivalent control, was mixed for 2 min (Velp Scientifica ZX3 Vortexer; 200 rpm), and then immediately added to the beaker. The system was left to settle for 48 h prior to adding three *C. volutator* per beaker (3 individuals per beaker and 5 beakers per treatment, n = 15). The experiment began once all *Corophium* were burrowed.

After 1 h, the experiment was terminated, and the individuals were removed from the beakers using a modified 3 mL Liquipette with the tip cut off. The *C. volutator* individuals were frozen at −20 °C prior to analysis. The fecal pellets were collected in the surface deposit feeding treatment by removing the sediment surrounding the burrow using a 3 mL Liquipette.

### 2.7 Tire Particles Identification

Tire particles were visually identified using criteria set out by Knight et al.^1^ Particles categorized as tire particles should (1) be black, (2) return to their original shape following compression, and (3) not break, crumble, or separate during compression. Use of controls and procedural blanks were used to account for any potential contamination. While the presence of microplastics can be validated using spectroscopic techniques (e.g., FTIR, Raman), this is not feasible for tire particles given their chemical composition and color providing poor spectra.

### 2.8 Tire Particle Adherence

To establish if tire particles adhered to the *C. volutator*, specimens were viewed under a microscope (Olympus SZX16, magnification: 2.52×). The abundance of tire particles on the carapace and appendages (gnathopods, pleopods, pereopods, and uropods (Figure 1)) was recorded. Images and measurements were taken using CellSens (Olympus). Once all the tire particles were captured, the organisms were rinsed ten times by bathing with Milli-Q water to ensure all adhered particles had been removed prior to digestion.

### 2.9 Tire Particle Ingestion

To determine if tire particles were ingested, *C. volutator* individuals were digested using 8% NaClO (GPR Rectapur, VWR Chemicals). In brief, *C. volutator* was added to 50 mL of NaClO and then placed in an orbital incubator (Sanyo, 120 rpm) at 50 °C for 16 h. The solutions were then vacuum filtered onto a 0.2 μm polycarbonate filter. Filters were visualized and enumerated under a microscope (Olympus SZX16, magnification: 11.59×) and CellSens (Olympus) was used to measure the maximal dimension of each tire particle. A procedural blank was included to provide certainty that the NaClO was not introducing particles that might be confused with tire particles.

### 2.10 Tire Particle Egestion

To understand if tire particles are retained within an organism once they have been ingested, the fecal pellets of *C. volutator* were checked visually for tire particles in an egestion experiment. *C. volutator* were exposed to tire particles in the same way to “Exposure 1” and “Exposure 2”, after the 1 h exposure, the individuals were added to a Petri-dish containing FSW and left to depurate for 1 h. The organisms were then removed, and visual checks were carried out under a microscope (Olympus SZX16) to confirm the presence of tire particles within fecal pellets. CellSens (Olympus) was used to image any fecal pellets containing tire particles.

### 2.11 Tire Particle Calculations

To establish how many tire particles were available to be ingested by the surface deposit-feeding individuals, we required the percentage of the sediment bioavailable to an individual *Corophium* and the total number of particles in the sediment. *Corophium* scrape particles from sediment around both ends of their U-shaped burrows, to an approximate distance of 4 mm (based on the average lengths of the second antennae of the tested *Corophium*). Using the formula 2 × πr^2^, this equates to 100 mm^2^ per individual, or 1% of the total surface area of sediment within each 112 mm diameter beaker. Tire concentrations by particle count (tire particle/kg or tire particle/L) were estimated from mass concentrations (g/kg or g/L) assuming the average mass of a worn tire particle is 0.29 μg.^39^ As such, the number of tire particles bioavailable to an *Corophium* was calculated as 46 particles/individual.

### 2.12 Statistical Analysis

Conformity to assumptions of distribution normality and homogeneity of variance were determined using a Shapiro-Wilk Test and a Levene’s Test where applicable. Negative binomial Generalized Linear Mixed Models were run for “ingestion” (glmer.nb­(particle.count ∼ Treatment + sex + (1|tank), data = df)) and “adherence” (glmer.nb­(particle.count ∼ Treatment + (1|tank), data = df)) data and selected based on the lowest AIC Value. To assess adherence of particles to body parts, a Kruskal–Wallis test was carried out followed by a pairwise Dunn’s test. Welsh two-sample *t* test and Wilcox tests were carried out where appropriate on tire particle length data for ingested and adhered particles between the two treatments. Significant difference was assigned where *P* ≤ 0.05. All statistics were conducted using R 4.3.0 ^67^ and tidyverse^68^ and graphs were produced using ggplot2.^69^

## 3 Results

### 3.1 Adherence of Tire Particles

Tire particles were adhered to all suspension-feeding and surface deposit-feeding *C. volutator* specimens analyzed (Figure 2). Pairwise comparisons using Dunn’s test show that there were more tire particles adhered to the surface deposit-feeding individuals compared to the surface deposit-feeding control individuals (Table 1). In the surface deposit-feeding treatment, the number of tire particles adhered ranged from 5 to 120 particles/individual, with a median of 20 particles/individual (Figure 3A). In the surface deposit-feeding control there was some evidence of tire particles adhering (0–16 particles/individual); however, Py-GC-MS did not detect any tire particles in the sediment (Table S1). Posthoc tests also revealed a significant difference in the number of tire particles adhering to individuals in the suspension-feeding treatment compared to the suspension-feeding control individuals (Table 1). The number of tire particles adhered to *C. volutator* in the suspension treatment ranged from 1 to 51 particles/individual, with a median of 19 particles/individual and there was no evidence of tire particles adhering to specimens in the suspension-feeding control. There was no significant difference in the number of tire particles that adhered to individuals between the suspension-feeding treatment and surface deposit-feeding treatment (Table 1, Table S2).

**Table 1:** Post-hoc Dunn’s Test Results for the Number of Tire Particles Adhered to Individuals among the Two Treatments and Controls^a^

Comparison	Z	P adjusted
Deposit vs Deposit Control	3.11	**0.01**
Deposit vs Suspension	–0.80	1.00
Deposit Control vs Suspension	–3.93	**<0.001**
Deposit vs Suspension Control	5.14	**<0.001**
Deposit Control vs Suspension Control	2.00	0.270
Suspension vs Suspension Control	5.94	**<0.001**

a Statistical significance was determined at *p* < 0.05.

**Figure 2:**
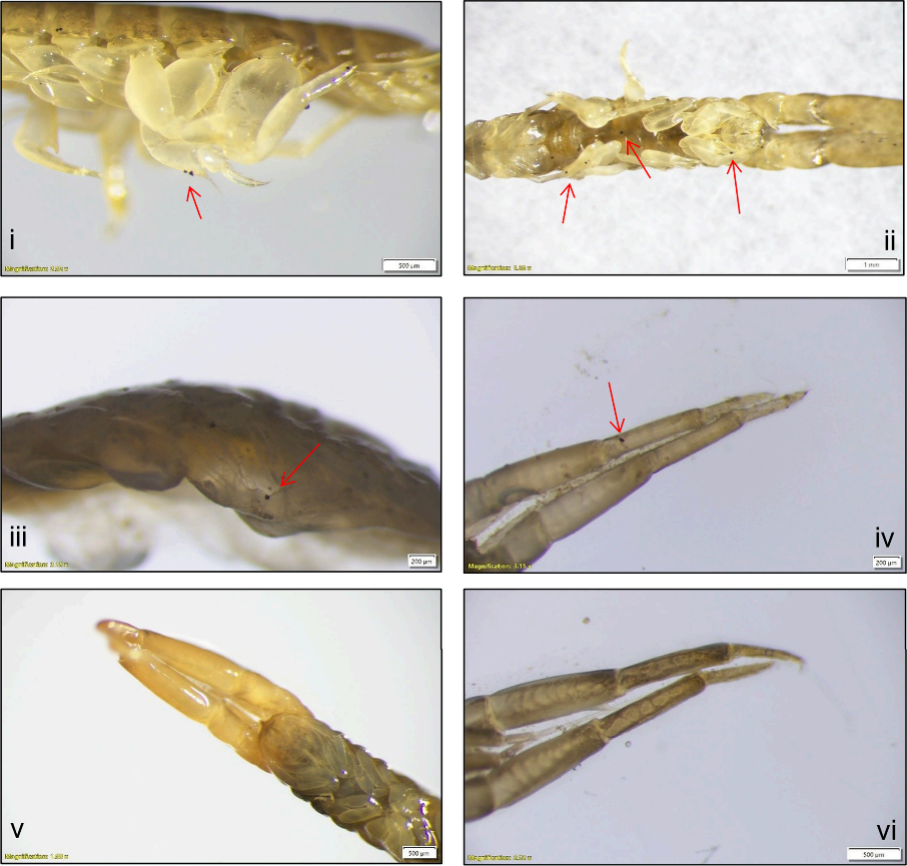
Tire particles adhered to a range of body parts of *C. volutator*, as visualized using a stereo microscopy (Olympus SZX16): (i) *C. volutator* periopods adhering tire particles in the suspension treatment (lateral view); (ii) *C. volutator* abdomen, gnathopods, and periopods adhering tire particles in the suspension treatment (ventral view); (iii) *C. volutator* periopods adhering tire particles in the surface-deposit treatment (lateral view); (iv) *C. volutator* 2nd antenna adhering tire particles in the surface-deposit treatment (ventral view); (v) *C. volutator* antenna and gnathopods in the suspension control treatment absent of tire particles (ventral view); and (vi) *C. volutator* antenna in the surface-deposit control treatment absent of tire particles (dorsal view).

**Figure 3:**
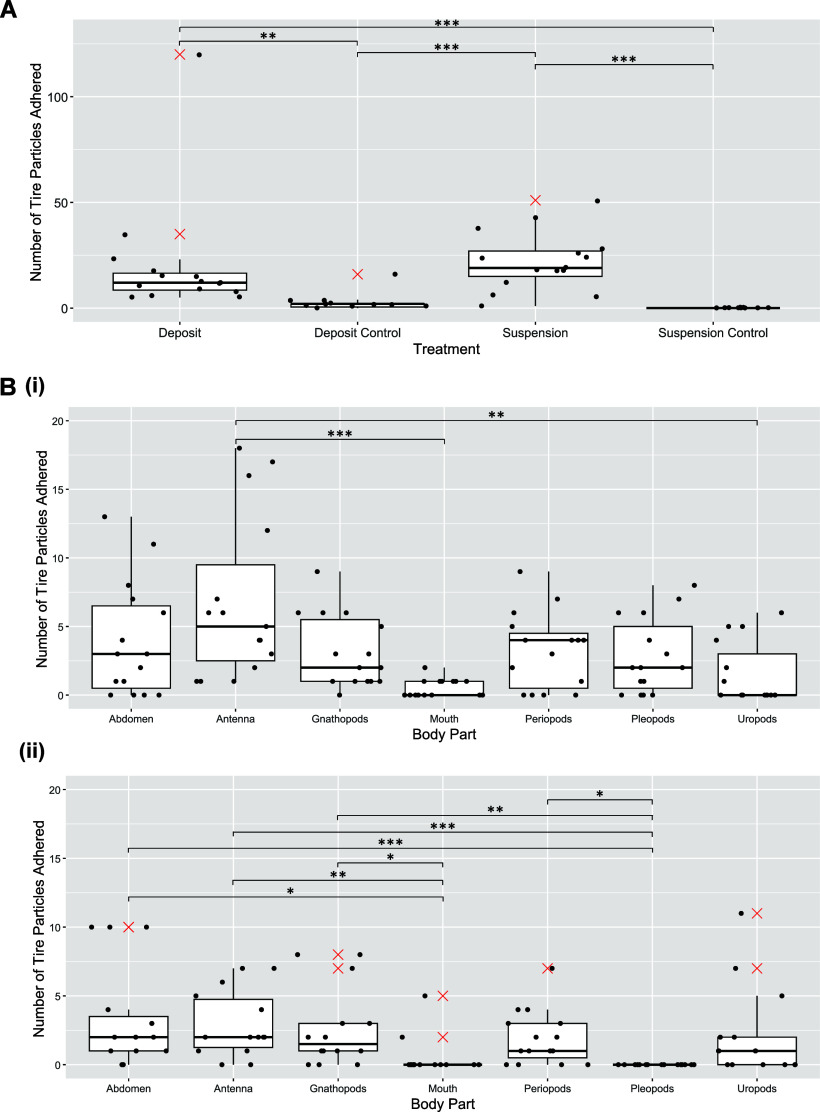
(A) Number of tire particles adhered to *C. volutator* in the deposit-feeding and suspension-feeding treatments. The middle line is the median, the box represents the interquartile range, and the whiskers are the 95% confidence limits. Black points are raw data, and the red cross indicates the outlier. (B) The number of tire particles adhered to each body part of *C. volutator* in the suspension (i) and surface deposit (ii) treatments. The middle line is the median, the box represents the interquartile range, and the whiskers are the 95% confidence limits. Black points are raw data, and the red cross indicates the outliers. Two data points in (ii) >30 were excluded from graph to improve comparability. Significance codes: *p < 0.05, **p < 0.01, ***p < 0.001.

Tires particles were observed to adhere to all body parts of *C. volutator* in the suspension treatment; however, there was a significant difference in where these particles aggregated (Kruskal–Wallis, df = 6, *p* < 0.001) (Figure 3B­(i)). The highest number of tire particles (n = 103) was found on the antenna and the lowest number was observed in the mouth (n = 8). Pairwise comparisons using Dunn’s test show that there are significantly more tire particles adhered to *C. volutator* antennae compared to the mouth (*p* < 0.001) and uropods (*p* < 0.01).

Similarly, tire particles were observed to adhere to all body parts of *C. volutator* in the surface-deposit treatment, with a significant difference of where these particles were adhering (Kruskal–Wallis, df = 6, *p* < 0.001) (Figure 3B­(ii)). The highest number of tire particles adhered was on the gnathopods (n = 115). Pairwise comparisons using Dunn’s test show that there are significantly fewer tire particles adhered to *C. volutator* pleopods compared to the abdomen (*p* < 0.001), antenna (*p* < 0.001) and gnathopods (*p* < 0.001). Also, the antenna had significantly more particles than the mouth (*p* < 0.01).

### 3.2 Ingestion of Tire Particles

Tire particles were ingested by all suspension-feeding and surface deposit-feeding *C. volutator* specimens analyzed. Individual *C. volutator* ingested between 102 and 267 tire particles in the suspension-feeding treatment; the mean number of tire particles present in the organism following an hour of exposure was 193 (Figure 4A). This was significantly different to the suspension-feeding control, with 0–5 particles/individual (GLMM, *p* < 0.0001). In the surface deposit treatment, individual *C. volutator* ingested between 2 and 20 tire particles, with the mean number of 10 tire particles ingested within an hour (Figure 4A). Tire particles were also identified in the surface deposit-feeding controls (0–2 particles/individual); this was significantly different from the surface deposit-feeding treatment (GLMM, *p* < 0.0001). There is a significant difference in the number of tire particles ingested between treatments, with organisms in the suspension treatment ingesting more particles than any other treatment (Table 2). In both treatments, male *C. volutator* individuals ingested significantly more tire particles than the females *C. volutator* in these treatments (Table 2).

**Figure 4:**
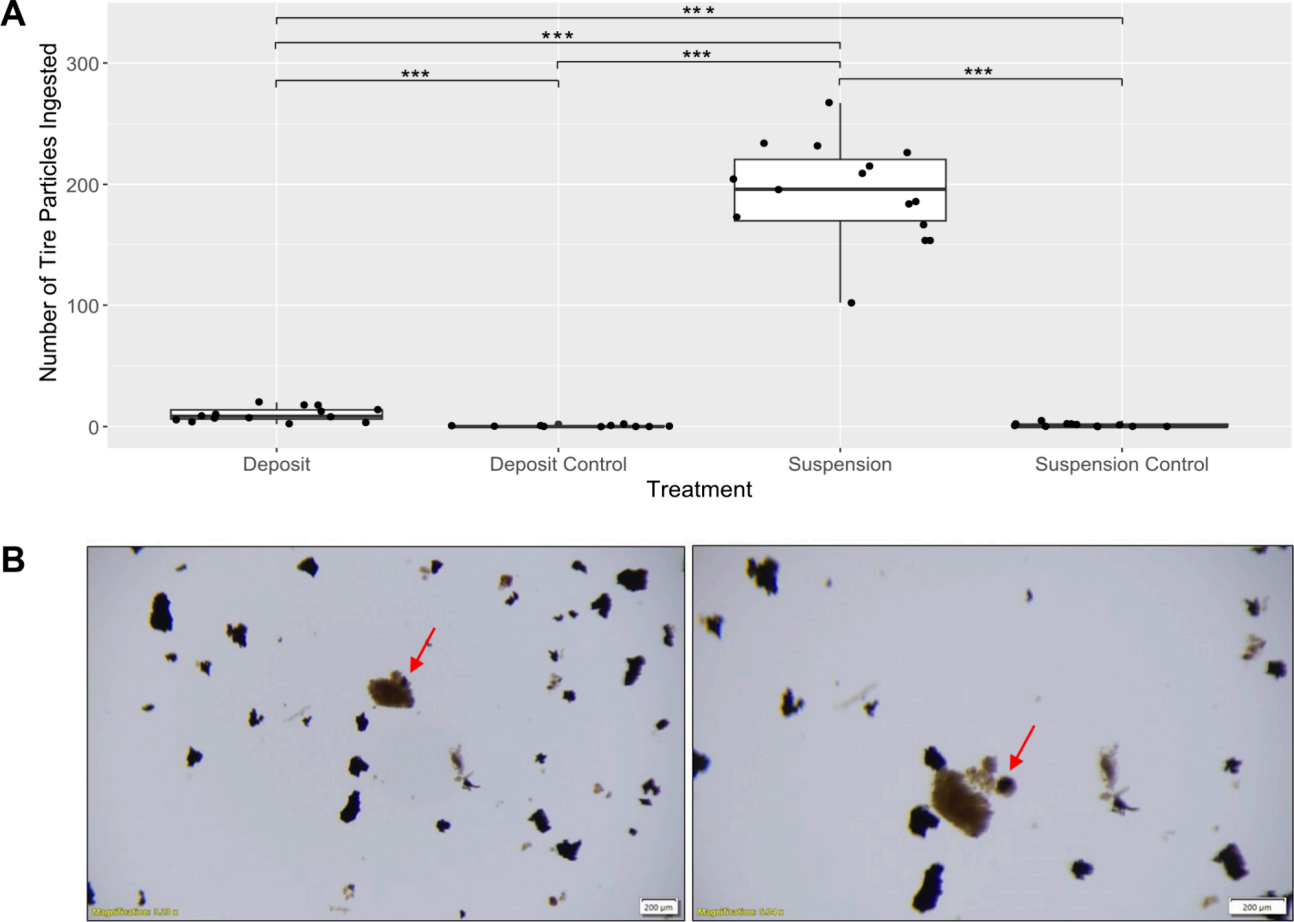
(A) Number of tire particles ingested by *Corophium volutator* in the suspension treatment. The middle line is the median, the box represents the interquartile range, and the whiskers are the 95% confidence limits. Black points are raw data. Significance codes: *p < 0.05, **p < 0.01, ***p < 0.001. (B) Fecal pellet from suspension-feeding *C. volutator* from the egestion study. Image on the left shows the fecal pellet containing a tire particle. Image on the right shows the same fecal pellet with the tire particle removed.

**Table 2:** Summary of Generalized Linear Mixed Model (GLMM) Results for the Ingestion Data

Fixed Effects				
	Estimate	Std. Error	z-Value	p-Value
Intercept	2.1275	0.1427	14.914	<0.0001
Treatment				
Deposit Control	–3.3829	0.4756	–7.113	<0.0001
Suspension	2.9739	0.1630	18.241	<0.0001
Suspension Control	–2.3398	0.3054	–7.661	<0.0001
Sex				
Male	0.2827	0.1051	2.689	0.0072

Random Effects		
Group	Variance	Std. Dev.
tank	0.03553	0.1885

#### 3.2.1 Egestion of Tire Particles

Tire particles were observed within fecal pellets of *C. volutator* individuals in both the suspension-feeding and surface deposit-feeding treatments from the egestion experiment (Figure 4B).

### 3.3 Length of the Tire Particles

The adhered tire particles were significantly larger than the ingested tire particles in the suspension treatment (Wilcox, W = 903769, *p* < 0.001) (Figure 5A).

**Figure 5:**
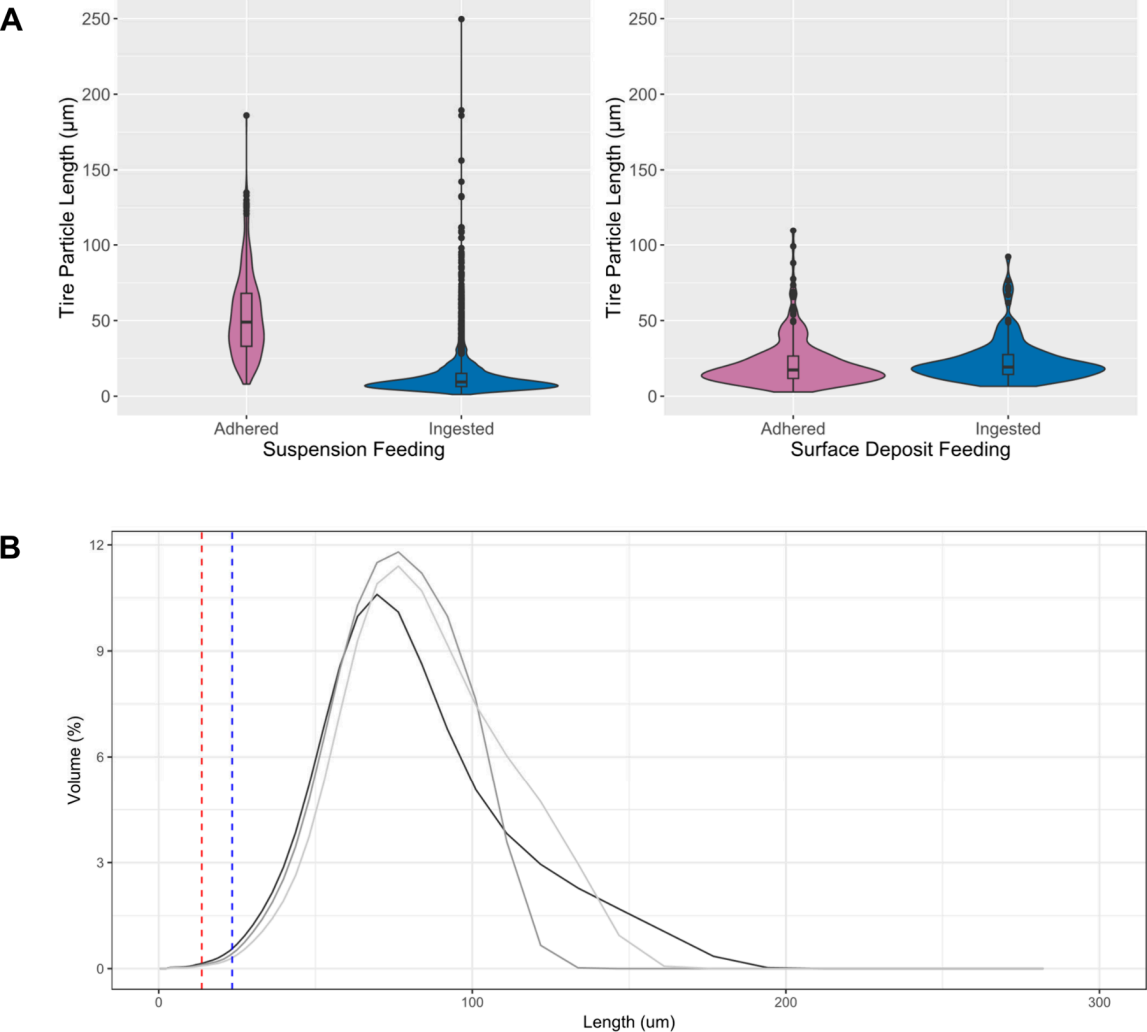
(A) Length of tire particles (μm) adhered and ingested by *C. volutator* in the suspension-feeding and deposit-feeding treatment. (B) Size distribution of tire particles added to the treatments. Each line is a replicate. The red dashed line is the average length of tire particles ingested in the suspension-feeding treatment. The blue dashed line is the average length of tire particles ingested in the surface deposit-feeding treatment.

There was no significant difference between the size of the adhered and ingested particles in the surface deposit treatment (Welch two-sample t (df = 321.8), t = −2.5021, p = 0.013; Figure 5A).

The adhered particles in the suspension feeding treatment are significantly larger than the adhered particles in surface deposit feeding treatment (Wilcox, W = 12301.5, p < 0.0001).

Feeding selectivity by *C. volutator* was observed as mean particle size of the available particles 71 μm, while the mean size ingested in the suspension-treatment was 14 μm and the surface deposit feeding-treatment was 23 μm (Figure 5B).

## 4 Discussion

With a growing concern for tire particles as an environmental pollutant, it is crucial to understand the extent to which organisms may be exposed to these anthropogenic particulates. The results of this study demonstrate that the estuarine amphipod *C. volutator* indiscriminately ingested tire particles irrespective of feeding mechanism. However, suspension feeding upon tire particles suspended in solution resulted in significantly greater amounts of particle ingestion. Tire particles adhered to all individuals, with particles accumulating on the antenna, gnathopods (feeding appendages), and abdomen. The majority of tire particles ingested and adhered by *Corophium* were <64 μm in length.

### 4.1 Ingestion of Tire Particles

Tire particles were ingested by all individuals, and *C. volutator* in the suspension-feeding treatment contained 19-fold greater amount of tire particles compared to the surface deposit-feeding individuals. Behavior studies of *C. volutator* observed surface deposit-feeding to be more selective as they sift through the sediment and reject inedible material using their second antennae.^57^ Whereas with suspension feeding, particles are pulled into the burrow via a feeding current and are captured on the gnathopods and ingested more indiscriminately.^60^ The number of ingested particles in the suspension may also be higher due to these tire particles being available for ingestion throughout the 1 h exposure. In the surface deposit feeding, given the feeding strategy for the *Corophium,* for the three individuals only 0.48% of the available tire particles would be bioavailable, equating to 46 tire particles/individual. Surface deposit feeding *Corophium* ingested ∼ 10 tire particles/individuals, approximately 22% of bioavailable tire particles.

The role of feeding mode in tire particle uptake is echoed in a number of recent studies. For example, when exposed to sediment dosed with 10% tire particles/kg (dw), the raptorial polychaete, *Hediste diversicolor* ingested 17 ± 6 tire particles/individual, where *Scrobicularia plana*, deposit feeders that nonselectively suck up surface sediment via a siphon,^70^ ingested 584 tire particles/individual.^42^ Uptake of tire particles has also been observed in freshwater organisms, *Gammarus pulex* was exposed to concentrations of tire particles at 3% dw in sediment and 10% dw in sediment. Following the exposure, *G. pulex* was found to have ingested an average of 2.5 tire particles/individual.^40^ A similar pattern has also been observed with microfibres where filter feeding *H. diversicolor* contained ∼15,000% more fibres than deposit feeding *H. diversicolor.* ^71^

In this study, *Corophium* were exposed to tire particles 0–150 μm in size but predominantly ingested tire particles 1–63 μm in size. This validates feeding studies that suggested *C. volutator* only ingest particles up to 63 μm.^60^ Size selectivity may result from the animals’ physiology, whereby the mouth gape limits ingestion of larger particles, or result from postcapture feeding selectivity which has been observed in other *Corophium* species.^72^ Ingestion of tire particles >64 μm may occur when particles are oriented in a particular way or represent smaller particles that were agglomerated in the gastrointestinal tract or amalgamated during the digestion and filtering processing steps. The morphological differences between the sexes may also explain why males ingested more tire particles in both the suspension-feeding and surface deposit-feeding treatments. Male *C. volutator* second antennas are thicker and longer than females, and therefore in the surface-deposit feeding treatment, they could pull down more tire particles into the burrow compared to the females and therefore ingest a greater number of tire particles. The size of the particles ingested will impact on their mode of action.^73^ For example, smaller particles (<20 μm) could translocate from the intestinal tract and directly interact with organs.^74^ Larger particles, or agglomerations of smaller particles, may cause blockages of the intestinal tract, potentially causing damage to tissues or limiting feeding capacity.^75,76^

### 4.2 Adherence of Tire Particles

In both the suspension and surface-deposit treatments, all individuals were found to have tire particles adhered to their external appendages. This is the first evidence that tire particles readily adhere to the carapace and appendages of biota. The antennae were found to adhere the highest amount of tire particles. When suspension feeding, water is drawn through the burrow via feeding currents generated by the gnathopods, and when deposit feeding, the antennae are used to scrape sediment into the burrow. The gnathopods were also found to have a slightly higher number of tire particles adhered compared to other body types; the gnathopods are feeding appendages covered in hair-like setae that entrap particles, which bring captured food to the mouth.^57^ As tire particles are hydrophobic, it may be hard for *C. volutator* to reject these particles once bound to the gnathopods and antennae. Similarly, Cole et al.^77^ showed polystyrene beads (0.4–3.8 μm) adhered to the external appendages of exposed copepods, with particles becoming entrapped between the setae of the swimming legs and feeding appendages. Bakelite microplastics have also been shown to adhere to the body of the water flea, *Daphnia magna* and fronds and roots of the aquatic plant, *Lemna minor.* ^78^ A difference in adherence between the two treatments is shown in the pleopods. In the deposit feeding treatment, the pleopods were not found to adhere any tire particles, while an average of 2.8 particles were adhered to the pleopods of *Corophium* in the suspension feeding treatment.

Given that adherence of microplastics has been observed in a number of organisms, it can be considered an important metric for understanding the interactions microplastics have with organisms and the environment. In this study, feeding mode had no impact on the number of tire particles adhered to individuals. However, *Corophium* in the suspension feeding treatment adhered larger particles compared to those in the surface deposit feeding treatment. It is unclear what is driving the particle size differences between feeding modes, although we postulate it may reflect the differential settling rates of tire particles of varying size and active irrigation of suspension feeding *Corophium* drawing in a broader size spectrum of tire particles, as compared with surface deposit feeding individuals.

Polysorbate was used in these exposures to mimic natural conditions. In the dynamic environment of estuaries tire particles are naturally mixed. However, in the experimental beakers the surface tension and hydrophobicity of the tire particles mean tire particles do not readily sink. Adding polysorbate as a surfactant ensured that tire particles behaved in a manner similar to how they would in the environment. As a surfactant, this may reduce adhesion between the particles and organisms limiting adherence,^79^ as such the results of this study could be considered an underestimate.

### 4.3 Ecological Relevance and Importance of the Study

The fate of adhered and ingested tire particles is currently unclear. Feeding studies with microplastics have shown a wide array of biota, including copepods, salps, and fish, will egest these anthropogenic particulates in their faeces.^77,80,81^ In this study, we similarly observed that, irrespective of feeding mode, ingested tire particles are subsequently egested within fecal pellets. Our observations of *C. volutator* indicate faeces are removed from the burrows, either via water current or biotic manipulation, and deposited on the nearby surface sediment. These faeces may become suspended in the water column resulting in their relocation, or buried into sediments via biotic and abiotic processes.^66,82^ Similarly, incorporation of tire particles into faeces may enhance uptake of the particulates by coprophagous organisms and detritivores, or those organisms that demonstrate food selectivity.^82,83^

Ingestion of tire particles may result in adverse health effects.^44^ For example, the Mexican scud (*Hyalella azteca)* showed a significant decrease in growth and reproduction and increased mortality when exposed to tire particles (<500 μm; ∼4.35 g/L).^39^ Furthermore, inland silverside (*Menidia beryllina*) demonstrated reduced growth when exposed to tire particles (0.0038 g/L).^47^ The mechanisms of toxicity are poorly elucidated, but like microplastics may stem from physical damage, leaching of toxic chemicals, or synergistic effects.^84,85^ Tire particles contain high concentrations of metals (e.g., zinc, lead) and chemicals (e.g., benzothiazole, phthalates) that can leach under environmental conditions.^37,44,86^ Tire leachates have been demonstrated to increase body length and width and decreased number of neonates in *Daphnia magna* from concentrations starting at 12.5 mg/L.^86^ Tire leachates have also been documented to cause increased mortality in a range of organisms from copepods to fish.^87,88^ *Corophium* represents a valuable test species for exploring the impacts of tire particles, with previous studies showing their sensitivity to a range of chemicals including copper, zinc and cadmium^89^ and toxicants such as crude oil.^61^ Their wide geographical distribution and sensitivity to toxicants has made *C. volutator* a standard test organism for sediment toxicity tests.^90^ Given *C. volutator* are ecologically important species that have an important role in estuarine food webs, any impacts of *Corophium* populations may have wider ecological implications including reducing sediment turnover^56^ and a reduction in prey for fish and wading birds.^54,91^ Furthermore, ingestion of tire particles will introduce metals and chemicals that might biomagnify in the food chain.^92^

This study provides the first evidence of the tire particle adhesion in biota. Adhesion of microplastics to biota has been documented both in the lab and field in a range of organisms, including microorganisms, plants, macroalgae, and animals.^78,93–95^ However, the effects of microplastic and tire particle adherence are largely unexplored. In the red coral, *Corallium rubrum*, adherence of microplastics was associated with evidence of tissue abrasion,^96^ while accumulation of macroplastics has been linked with increased susceptibility to disease in corals.^97^ The adherence of tire particles on the body surface extends the exposure period, increasing the likelihood of metals and toxic organic compounds to leach from the particle into the organism. Our study found the highest number of tire particles were adhered to the antennae. The antennae are used to sense their environment and aid in crawling behavior.^57^ As such, we postulate tire particle adherence could affect chemoreception and motility, with repercussions at the population level (Figure 6). For example, reduced ability to detect food or predators may lead to population decrease, while reducing movement and feeding can reduce sediment turnover essential to nutrient cycling.^58^ Reduced motility and chemoreception could also reduce the number of males leaving their burrows to seek out females by following pheromone trails,^98^ leading to reduced reproduction and population size. Tire particles may also interfere with moulting, with prior studies evidencing that microplastics can affect moulting in coldwater copepods^99^ and heavy metals can inhibit moulting in the crab *Chasmagnathus granulata.* ^100^ Moulting plays a key role in growth and development of a wide array of species, and also plays a role in *Corophium* reproduction, as females are fertilized immediately following a moult.^91,101,102^ As such, feeding, growth, and reproduction are recommended as biomarkers of health in future, longer-term exposure studies.

**Figure 6:**
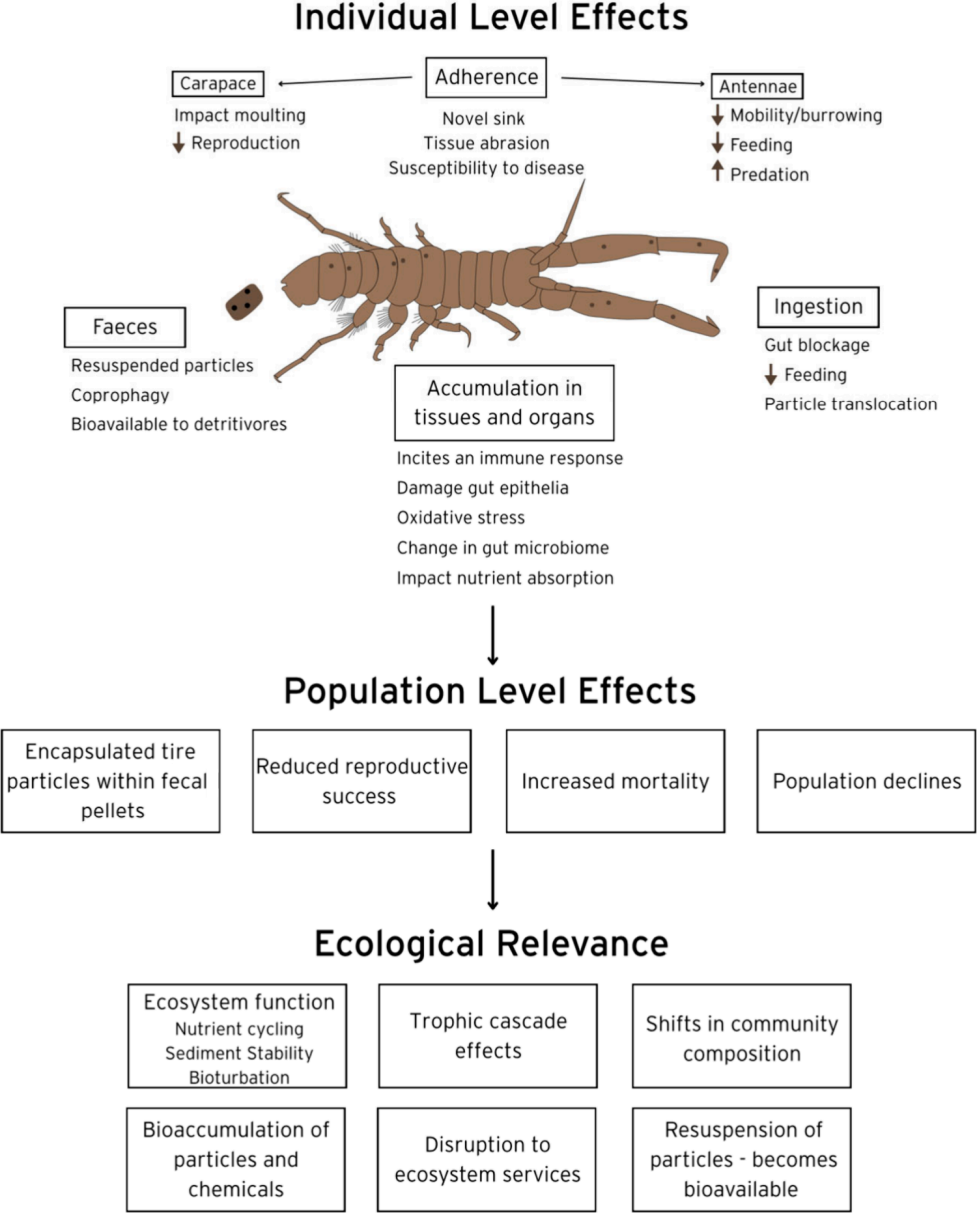
Postulated effects of tire particles at individual and population levels with ecological relevance.

Exposed to environmentally relevant concentrations of tire particles for 1 h, *C. volutator* were observed to ingest and adhere tire particles. Tire particles predominantly adhered to the antenna irrespective of feeding mode. Suspension feeding resulted in the uptake of 102–267 tire particles/individual. Deposit-feeding resulted in the uptake of 2–20 tire particles, although it is important to note that when deposit feeding, the *Corophium* would only have had access to <5% of the available tire particles given their sedentary lifestyle. The short exposure time (1 h) was selected to observe the immediate interactions between tire particles and the organisms in the different feeding modes. Tire effluent from roads is discharged during rain events and on average, these events last 1.5 h.^30^ Laboratory settling studies have observed over 85% of tire particles settle after an hour,^31^ therefore, for suspension feeding organisms, this is the time frame where tire particles are most likely to be bioavailable. Further work is required to understand the fate and toxicity of tire particles to exposed biota, for which chronic exposure studies are recommended. Interactions were observed at concentrations of 0.1 g/L and 0.1 g/kg, whereas concentrations of 0.0–1.6 g/kg^28,29^ have been observed in estuarine habitats. As such, adherence and ingestion of tire particles can be expected to be commonplace in estuaries where tire particles and *Corophium* co-occur. Given, environmental tire particle concentrations are expected to increase owing to an increasing global population and the rise of heavier electric vehicles it is vitally important that we understand how such anthropogenic particles may enter marine food webs and scope their toxicological risk.

## Supplementary Material



## Notes

Size fraction of tire particles; sampling site map; tire particle sediment concentrations; and summary of Generalized Linear Mixed Model (PDF)
