# Supplementary material for: Comparison of the Uptake of Tire Particles via Suspension and Surface Deposit Feeding in the Estuarine Amphipod Corophium volutator
Source: Environ Sci Technol. 2025 Aug 20;59(34):18259–71. doi: 10.1021/acs.est.5c03654 (PMC12409897; doi:10.1021/acs.est.5c03654)
Supplement: Supplementary file 1 [file es5c03654_si_001.pdf]

Supplementary Information

A comparison of the uptake of tyre particles via suspension and surface deposit feeding in the estuarine amphipod *Corophium volutator*

Charlotte Woodhouse[1, 2], Penelope Lindeque[1], Tamara Galloway[2], Geoffrey D. Abbott[3], Matthew Cole[1]

[1]Plymouth Marine Laboratory, Prospect Place, The Hoe, Plymouth, PL1 3DH, UK
[2]University of Exeter, Geoffrey Pope Building, Stocker Road, Exeter, EX4 4QD, UK
[3]Newcastle University, Drummond Building, Newcastle upon Tyne, NE1 7RU, UK

*Corresponding author. Email address: mcol@pml.ac.uk

Supporting information:

Includes 3 pages, 2 figures and 2 tables

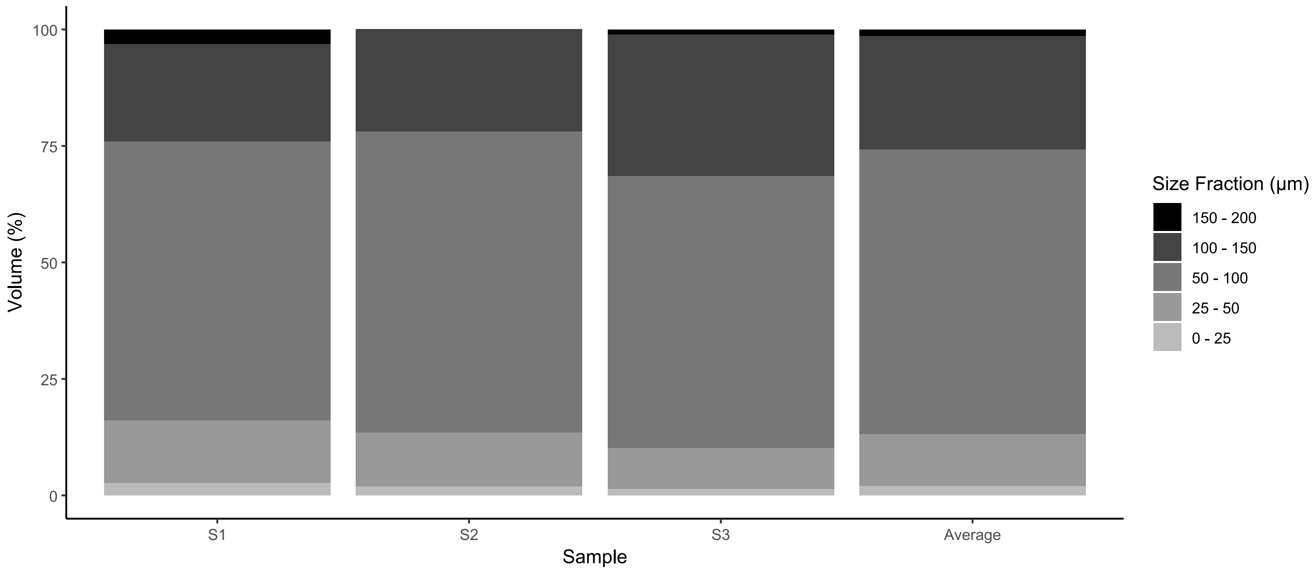

Figure S1. The percentage proportion of tyre particles within each size fraction added to the treatments. Each stacked bar chart is a replicate with the last bar chart showing the average proportion across the three replicates.

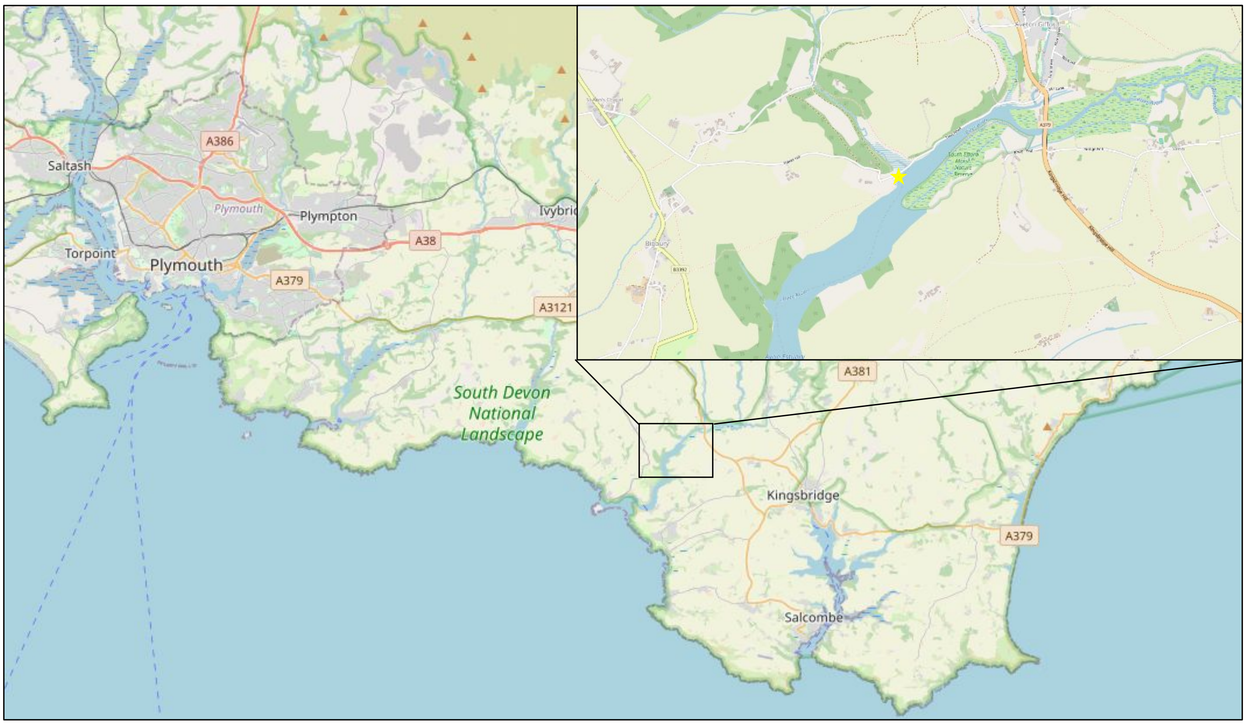

Figure S2. Map of Plymouth with inset map of Avon Estuary. Orange lines depict major roads (A roads) and yellow lines depict minor roads (B roads) and white lines depict all other roads (excluding motorways). The yellow star is where samples were taken. Image Credit: OpenStreetMap (www.openstreetmap.org).

Table S1. Concentration of benzothiazole (marker for tyre particles) in sediment from the sample site.

Sample ID	Mass Sample (mg)	Benzothiazole (ng/mg)	
Tidal Road-1	2.45	0	Not Detected
Tidal Road-2	2.50	0	Not Detected
Tidal Road-3	2.07	0	Not Detected

Table S2. Summary of Generalized Linear Mixed Model (GLMM) Results for the adherence data.

Fixed Effects	Estimate	Std. Error	z-value	p-value
Intercept	2.859	0.239	11.996	<0.001
Treatment: Deposit Control	0.1724	0.3369	0.512	0.609
Treatment: Suspension	0.1724	0.3369	0.512	0.609
Treatment: Suspension Control	-30.45	253600	0.000	1.000

Random Effects:	Group	Variance	Std. Dev.
	tank	0.1212	0.3481
